# Hemorrhagic complication of arachnoid cyst: A case report and literature review

**DOI:** 10.1016/j.radcr.2025.03.072

**Published:** 2025-04-17

**Authors:** Azad Star Hattam, Soran H. Tahir, Zana Omar Kak Abdullah, San Khasraw Mohammed, Hawkar A. Nasralla, Sanaa O. Karim, Berun A. Abdalla, Hawar A. Sofi, Sarhang Sedeeq Abdalla, Fahmi H. Kakamad

**Affiliations:** a Scientific Affairs Department, Smart Health Tower, Madam Mitterrand Street, Sulaymaniyah, Iraq; b College of Medicine, University of Sulaimani, Madam Mitterrand Street, Sulaymaniyah, Iraq; c College of Nursing, University of Sulaimani, Madam Mitterrand Street, Sulaymaniyah, Iraq; d Kscien Organization for Scientific Research (Middle East Office), Hamdi Street, Azadi Mall, Sulaymaniyah, Iraq

**Keywords:** Arachnoid cyst, Craniotomy, Cyst fenestration, Intracystic hemorrhage, Subdural hematoma

## Abstract

Arachnoid cysts (ACs) are congenital malformations that can form anywhere in the subarachnoid space along the cerebrospinal axis. While intracystic hemorrhage and subdural hematoma (SDH) are rare, they can be urgent complications that may require emergency craniotomy. This report aims to present a case of an AC complicated by intracystic hemorrhage and SDH. A 63-year-old man presented after a fall, reporting a mild headache, right-sided weakness, confusion, speech difficulties, and gait ataxia. Brain magnetic resonance imaging (MRI) revealed an acute or early subacute SDH. A cystic structure measuring 9 × 6 × 5 cm and showing hemorrhagic characteristics was identified deep within the left SDH. The patient underwent a left-sided craniotomy to evacuate the SDH and remove all surrounding membranes. Additionally, an intracystic hematoma was evacuated, and the cyst wall was fenestrated. The patient remained stable during the follow-up. Over the past decade, a brief literature review on AC complicated by intracystic hemorrhage and SDH identified 9 case reports encompassing ten cases. Patient ages ranged from 6 to 47 years, with only 2 (20%) female patients. Headaches were the most frequent symptom, present in all patients, while eight patients (80%) had no history of head trauma. Only two cases (10%) were treated conservatively. In conclusion, individuals with AC are vulnerable to developing intracystic hemorrhage and SDH, either spontaneously or post-trauma. Open craniotomy combined with cyst fenestration can lead to preferred outcomes in treating this condition.

## Graphical abstract

**Figure:**
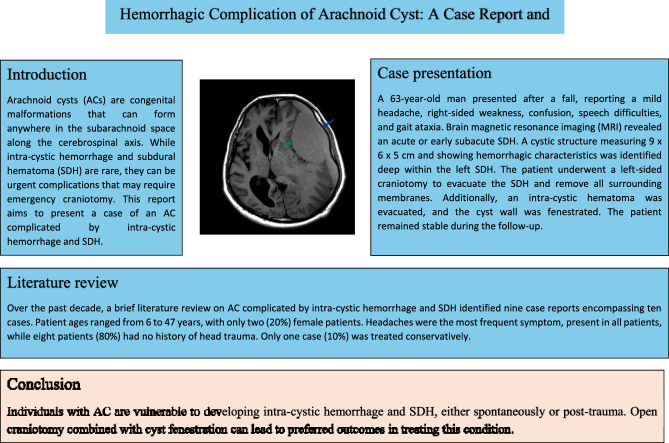

## Background

Intracranial arachnoid cysts (ACs) are noncancerous congenital structures filled with cerebrospinal fluid (CSF) that can potentially increase the risk of developing subdural hematomas (SDHs) following head injury [1]. These cysts are present in 2.6% of children and 1.4% of adults [1]. The ratio of males to females with this condition is 2:1 [2].

The ACs represent approximately 1% of all intracranial space-occupying lesions and are primarily found in the temporal fossa and Sylvian fissure [3]. They are generally asymptomatic and slow-growing, and their prevalence has risen in recent decades due to the more frequent use of brain imaging in routine clinical practice [4].

While most ACs remain stable throughout a person's life, they can occasionally undergo spontaneous changes such as complete disappearance, intracystic hemorrhage, enlargement, or rupture, leading to SDH [4]. These changes may occur naturally or following exertion, physical activity, or trauma [4]. Head trauma is considered one of the most significant risk factors for the development of intracystic hemorrhage in ACs and associated SDH [3].

Intracystic hemorrhage and SDH are rare but potentially urgent complications of ACs that may necessitate an emergency craniotomy [5]. This report describes a case of AC complicated by intracystic hemorrhage and SDH. It is structured according to the CaReL guidelines (a consensus-based framework designed to enhance the quality of clinical case reports by integrating detailed literature reviews), and all references have been reviewed for eligibility [6,7].

### Case presentation

**Fig. 1:**
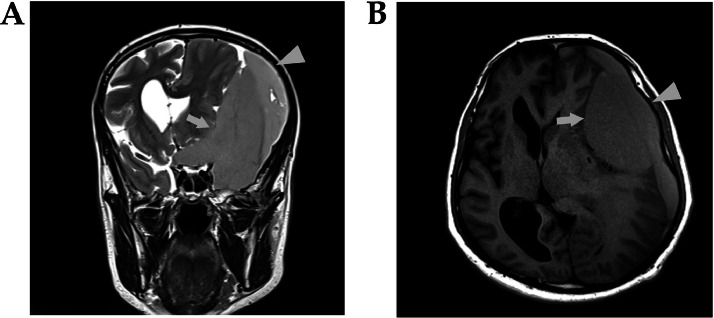
The brain MRI images demonstrate (A) A T2-weighted coronal section and (B) A T1-weighted axial section, revealing an intermediate signal collection on the left side comprising 2 components with varying gray levels. The “arrowhead” highlights the subdural collection, and the “ arrow” marks the cystic collection extending into the suprasellar region.

**Fig. 2:**
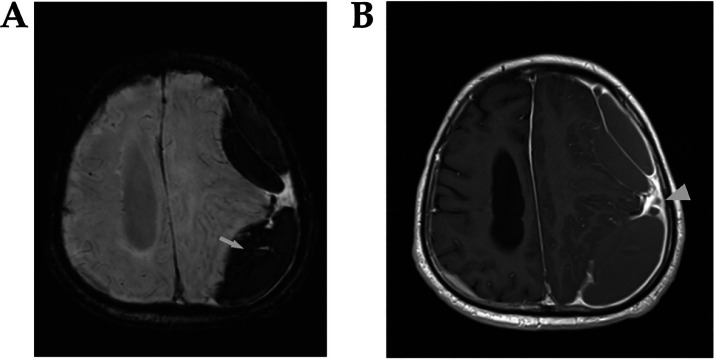
(A) The brain MRI susceptibility weighted imaging sequence axial section reveals a linear blooming signal, indicated by the “arrow,” which suggests hemorrhagic content. (B) The T1-weighted axial section with IV contrast displays dura enhancement, marked by the “arrowhead,” surrounding both the subdural collection and the cystic lesion.

**Fig. 3:**
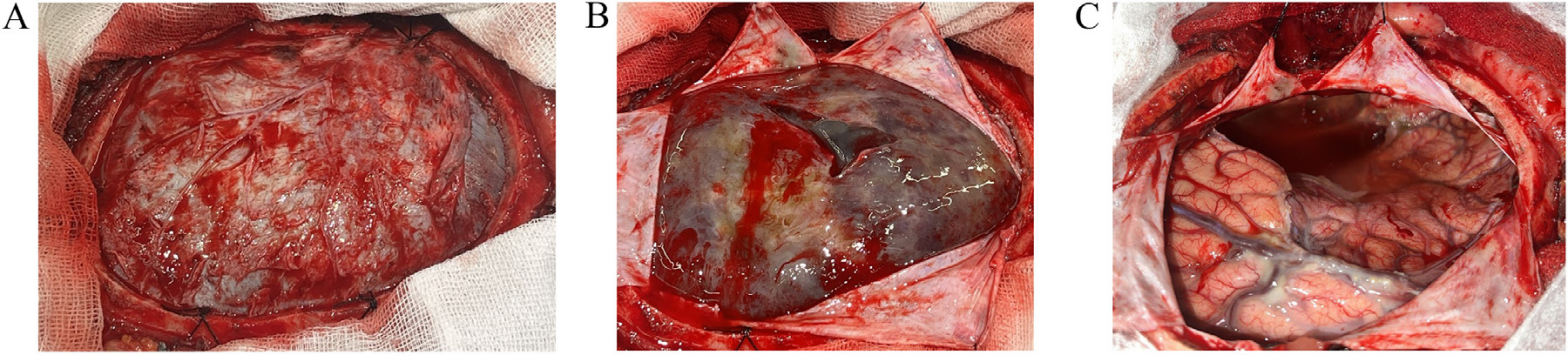
The intraoperative images demonstrate (A) A wide craniotomy with exposed dura, showing a dark appearance of the underlying hematoma. (B) A cruciate durotomy revealing a thick-walled SDH. (C) Complete removal of the SDH, exposing a clear cavity of the AC and the cortical surface of the brain, extending to the skull base following excision of the AC wall.

A 63-year-old man experienced a fall to the ground and presented with mild headache, right-sided weakness, confusion, difficulty speaking, and gait ataxia. There were no reports of vomiting or seizures. His medical and surgical history was unremarkable, and he had no history of relevant medications. On clinical examination, the patient was conscious and oriented, presenting with dysphagia, right-sided weakness, a positive Hofmann's sign on the right side, and an unsteady gait with no diplopia. Brain magnetic resonance imaging (MRI) was performed using a Siemens Magnetom Aera 1.5T scanner. Imaging sequences included T1-weighted imaging (T1WI), T2-weighted imaging (T2WI), fluid-attenuated inversion recovery (FLAIR) with fat suppression, susceptibility-weighted imaging (SWI), T1WI with fat suppression, and T1WI with intravenous contrast enhancement. The scan revealed a significant left-sided subdural collection, characterized by intermediate signal intensity on both T1WI and T2WI, with no diffusion restriction observed on diffusion-weighted imaging (DWI) (Fig. 1). Enhancement was noted in the surrounding dura mater. The collection extended from the left frontal to the occipital region and measured approximately 4 cm in thickness. These findings were indicative of an acute or early subacute SDH. Additionally, a cystic structure measuring 9 × 6 × 5 cm, exhibiting hemorrhagic signal characteristics, was observed deep in the left SDH. The hemorrhage was hyperintense to the cortex on T1WI and iso-hyperintense to the cortex on T2WI (Fig. 2). This structure extended into the suprasellar region and suggested an AC complicated by hemorrhage. All laboratory tests were normal except for a hematocrit of 32% (reference range: 38.8%-50%), and bleeding disorders were excluded. A left-side craniotomy was performed to remove the SDH, including all surrounding membranes (Fig. 3). The intraoperative findings revealed a thick blood clot on the cortex with an intracystic extension. The intracystic hematoma was also evacuated, and the cyst wall was fenestrated. After the operation, the patient showed notable improvements in motor strength and speech and was discharged 3 days later. At the 5-month follow-up, the patient remained stable and exhibited no signs or symptoms of the condition.

## Discussion

The natural history of ACs remains inadequately defined. These cysts originate from embryonic developmental anomalies, specifically the duplication or splitting of the arachnoid membrane. The ACs account for approximately 1% of all nontraumatic intracranial masses [8].

The most prevalent location for this etiology is the Sylvian fissure, observed in 49% of cases, followed by the cerebellopontine angle (11%), supracollicular region (10%), vermian area (9%), and sellar or suprasellar regions (9%) [8]. Other less common sites include the interhemispheric region (5%), cerebral convexity (4%), and clival region (3%) [8]. The current report noted that the AC was located deep inside the left SDH and extended into the suprasellar area.

AC wall consists of multiple layers of collagen-anchored arachnoid cells; minor head trauma, intense breathing, physical activities, or even coughing can lead to its rupture [1]. The primary risk factor associated with intracystic bleeding is head trauma [2]. Spontaneous hemorrhage is rarely reported in the literature [2]. Patients with ACs may benefit from avoiding sports or activities that pose a higher risk of head trauma. Although few patients with these lesions develop symptoms or require surgery, there is a risk of hemorrhage into the cyst or surrounding brain tissue. This risk should be considered when advising patients about the potential clinical progression of their condition [10]. The case described in the present report experienced a fall to the ground before being transported to our hospital.

Most ACs remain asymptomatic; however, in some cases, ACs may present with symptoms such as headaches, increased head circumference, and developmental delays in pediatric patients [5]. Rarely, they can also cause weakness, seizures, or psychiatric changes due to complications such as intracystic hemorrhage or SDH, which are rare, life-threatening, and unpredictable [2]. The patient described in this report was asymptomatic before the trauma. However, following the incident, the patient developed a mild headache, right-sided weakness, confusion, difficulty speaking, and gait ataxia.

ACs are typically found incidentally on cerebral imaging, appearing as hypo-intense masses outside the brain parenchyma. They are also sometimes identified during autopsies [2]. The case described in this report was diagnosed by a brain MRI after the incident.

AC is recognized for causing both chronic and acute SDH, particularly following trauma [9]. Although the pathophysiology of intracystic hemorrhage or SDH is not fully understood, two theories regarding hematomas have been proposed [1]. One theory suggests that small vessels between the dura mater and the AC may rupture, leading to intracystic bleeding or SDH [1]. Additionally, because the AC is less compliant than normal brain tissue, bridging veins or unsupported vessels over the cyst wall are particularly susceptible to hemorrhage and rupture [1]. According to a second theory, the AC wall secretes fluid, gradually increasing intracystic pressure [1]. This elevated pressure can cause the wall to rupture in areas devoid of vessels, forming subdural hygroma and SDH [1]. The AC of the patient described herein was complicated with intracystic hemorrhage and SDH after the trauma.

A brief literature review [[1], [2], [3], [4], [5],[8], [9], [10], [11]] on AC complicated by intracystic hemorrhage and SDH over the past decade revealed only 9 case reports involving ten cases. The ages ranged from 6 to 47 years, with only 2 (20%) female patients. Headaches were the most common symptom, affecting all patients, and eight patients (80%) had no history of head trauma. Only two cases (20%) were managed conservatively (Table 1).

**Table 1:** Review of recent literature from the past decade on cases involving intracystic hemorrhage in arachnoid cysts associated with subdural hematoma.

Author, year [reference]	Type of Study	Age (y)	Gender	Complaint	Hx of trauma	Diagnosed by	GCS	Location of AC	Treatment	Complication	Last Follow-up	PO patient status
Hanai et al. 2023 [1]	Case report	18	Male	mild headache	Yes	CT scan	N/A	Left convexity	Conservative treatment	No	6 m	Stable
Loiseau et al. 2021 [2]	Case report	23	Male	head trauma	Yes	CT scan	4/15	Rt Side of the brain	Craniotomy	Intracranial hypertension	40 d	Passed away
Aydogmus et al. 2017 [3]	Case report	15	Male	headache, nausea	No	CT scan	15/15	Lt temporal Sylvian	Burr hole drainage	No	N/A	Stable
Adin et al. 2018 [4]	Case report	36	Male	headache, vomiting, visual acuity	No	CT scan	15/15	Rt middle cranial fossa	Rt side craniotomy	No	1 y	Stable
		21	Male	severe headache	No	CT scan	15/15	Lt middle cranial fossa	Conservative treatment	No	6 m	Stable
Johnson et al. 2018 [5]	Case report	29	Female	progressive bifrontal headache	No	CT scan	N/A	Lt cerebral convexity	Lt side craniotomy	No	2 m	Stable
Kahiloğulları et al. 2014 [8]	Case report	6	Male	headache, vertigo, vomiting, somnolence	No	MRI, CT scan	Stable	Lt Middle cranial fossa	Lt frontotemporoparietal craniotomy	N/A	N/A	Stable
Kaszuba et al. 2018 [9]	Case report	47	Male	progressive headache, dizziness, unsteady gait, nausea, emesis	No	MRI, CT scan	N/A	Middle cranial fossa	Craniotomy	No	NA	Lt side Headache
Özkaçmaz et al. 2017 [10]	Case report	42	Male	headache, nausea	No	CT scan	N/A	Lt middle cranial Fossa	Frontotemporal craniotomy	N/A	N/A	N/A
Kieu et al. 2021 [11]	Case report	33	Female	headache	No	CT scan	N/A	Middle cranial fossa	Temporal craniotomy	No	1 y	Stable

Abbreviations: AC, arachnoid cyst; CT scan, computed tomography scan; GCS, Glasgow Coma Score; Hx, history; Lt, left; MRI, magnetic resonance imaging; N/A, nonapplicable; PO, postoperative; Rt, right; y, year; m, months; d, day.

Complications related to ACs include their potential for ongoing growth, which may exacerbate headaches and lead to focal neurological deficits [5]. In particular, suprasellar and quadrigeminal ACs can cause obstructive hydrocephalus or compress the brainstem, potentially resulting in life-threatening conditions [5].

Treatment options for ACs with SDH include open craniotomy with cyst fenestration, cystoperitoneal shunt, endoscopic cyst fenestration, burr hole drainage, and hematoma drainage without addressing the cyst itself. However, if the cyst is not fenestrated, completely opened, or removed, there may be a higher likelihood of a hematoma recurrence [9]. Although it is frequently controversial, recent reports suggest a generally benign natural progression, supporting a conservative management approach for children and adults [9]. Adin et al. described 2 adult cases of intracranial ACs with spontaneous intracystic hemorrhage and accompanying SDH, one of which was effectively managed with conservative treatment [4]. In the present report, a left-side craniotomy was performed to remove the SDH, including all surrounding membranes. Additionally, an intracystic hematoma was evacuated, and the cyst wall was fenestrated.

A limitation of this report is the lack of post-treatment imaging, as the patient refused to undergo any further imaging procedures.

## Conclusion

Individuals with AC are vulnerable to developing intracystic hemorrhage and SDH, either spontaneously or post-trauma. Open craniotomy combined with cyst fenestration can lead to positive outcomes in treating this condition.

## Consent for publication

Not applicable.

## Availability of data and material

All data and materials are kept by the first and corresponding authors.

## Patient consent

Consent has been taken from the patients and the family of the patients.
